# Acute Generalized Exanthematous Pustulosis: A Case Report

**DOI:** 10.7759/cureus.89576

**Published:** 2025-08-07

**Authors:** Hiebda Sofía Martínez Jiménez

**Affiliations:** 1 Internal Medicine, Centro Médico Nacional Siglo XXI, Mexico City, MEX

**Keywords:** acute generalized exanthematous pustulosis, clinical dermatology, dermatology, suspected drug eruption, symmetrical drug-related intertriginous and flexural exanthema

## Abstract

Acute generalized exanthematous pustulosis (AGEP) is a rare, potentially severe cutaneous adverse reaction characterized by the rapid onset of numerous small, sterile pustules on edematous erythema, commonly accompanied by systemic symptoms such as high-grade fever and neutrophilic leukocytosis. AGEP is most frequently triggered by medications, especially antibiotics, though infections and other exposures can also be causative. We report the case of a previously healthy 27-year-old male patient who developed a febrile pustular eruption with systemic involvement. Initial symptoms included sore throat, malaise, and a progressing maculopapular rash evolving into pustular plaques. The patient had previously taken oral ibuprofen without any adverse reactions; therefore, a drug-related trigger was considered unlikely, and an infectious etiology was suspected. Diagnosis of definite AGEP was established through clinical evaluation, laboratory findings showing leukocytosis with neutrophilia, and histopathology revealing subcorneal neutrophilic pustules with dermal edema and eosinophilic infiltrate. This case underscores the importance of early recognition of AGEP in febrile pustular eruptions, even without clear drug exposure.

## Introduction

Acute generalized exanthematous pustulosis (AGEP) is a rare and potentially severe cutaneous adverse reaction, marked by the sudden appearance of numerous small, non-follicular, sterile pustules on a background of edematous erythema. AGEP primarily affects adults, with a median age of around 60 years, and shows a female predominance [1]. 

AGEP is a T cell-mediated disease [2]. The eruption typically occurs in association with systemic symptoms, most notably fever, neutrophilic leukocytosis, elevated C-reactive protein (CRP), and, in one-third to half of cases, mild eosinophilia [1]. AGEP is most commonly triggered by medications, particularly antibiotics, but can also be associated with infections and other exposures [1,3]. The European Academy of Allergy and Clinical Immunology (EAACI) recently issued a position statement highlighting that AGEP should be considered when symptoms appear within one to 12 days after starting the suspected agent, typically within one to two days for antibiotics and seven to 12 days for other drugs [4].

The main differential diagnosis of AGEP is generalized pustular psoriasis. Histology typically shows spongiform pustules located subcorneally or within the epidermis, along with papillary dermal edema and a perivascular inflammatory infiltrate composed mainly of neutrophils and occasional eosinophils [1]. 

If a drug is identified as the triggering factor, it should be discontinued immediately. Treatment involves supportive measures such as skin care, emollients, and adequate hydration. The condition generally has an excellent prognosis, with mortality reported in less than 5% of cases [1,5]. Given its clinical overlap with other severe cutaneous adverse reactions, early recognition is crucial to guide appropriate management and avoid unnecessary treatments. We present the case of a previously healthy 27-year-old male patient who developed an AGEP. Laboratory tests showed marked neutrophilia and elevated inflammatory markers, and histopathology confirmed the diagnosis of AGEP. Early recognition and management were crucial for clinical improvement and resolution.

## Case presentation

**Figure 1:**
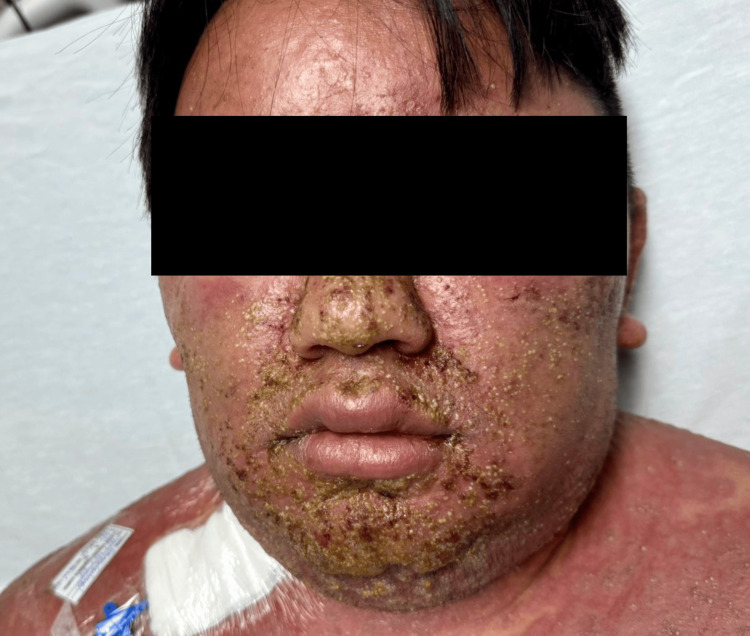
Facial involvement in acute generalized exanthematous pustulosis Multiple sterile pustules on an erythematous and edematous base, some coalescing and partially covered with meliceric crusts. Note: The patient consented to the use of their images in an open-access journal, and a written and signed consent statement from the patient was provided to the journal.

A 27-year-old male patient, with no significant past medical history, previously healthy, and with no known drug allergies, presented with a sore throat, general malaise, asthenia, and fatigue. Subsequently, 24 hours later, he developed facial swelling and a dermatosis on the face and anterior thorax, characterized by non-pruritic erythematous maculopapular lesions (Figure 1). He sought medical attention and was prescribed oral ibuprofen at an unknown dose. He took a single dose approximately six hours after symptom onset. Due to a lack of clinical improvement, he presented to a hospital around 14 hours after the initial facial swelling and skin eruption. Physical examination revealed a hyperemic pharynx and a generalized maculopapular exanthem. During his stay at that facility, he developed a persistent fever (>38.3ºC), and the dermatosis continued to progress in a cephalocaudal pattern, involving the genital area while sparing the palms. Given the persistence of skin lesions, the patient was referred for evaluation at a tertiary care center approximately 72 hours after the initial skin eruption.

**Figure 2:**
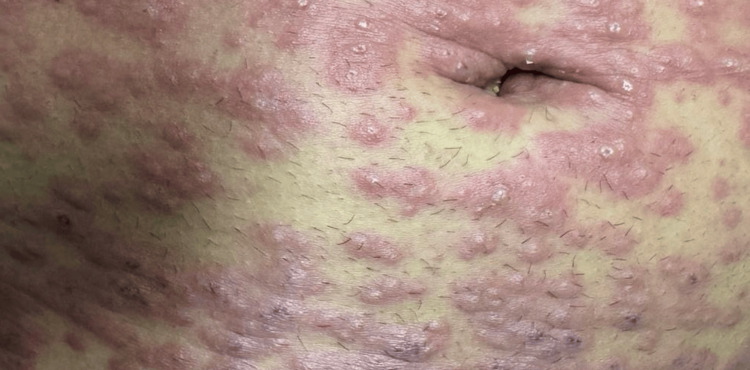
Abdominal pustular eruption in acute generalized exanthematous pustulosis

**Figure 3:**
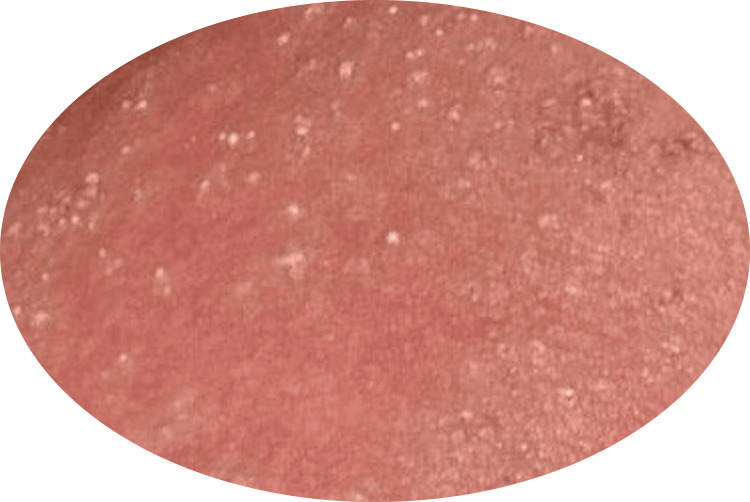
Pustules on a background of edematous erythem Clinical image of the thorax showing pustules on a background of edematous erythema, a characteristic feature of acute generalized exanthematous pustulosis as described in the introduction of this report.

Clinically, the presentation was compatible with AGEP (Figures 2, 3).

Thus, a skin biopsy was performed. At presentation, blood cultures were obtained; both central and peripheral cultures grew *Staphylococcus epidermidis*, which was considered a contaminant. Recommended tests, including complete blood count with differential (neutrophils, eosinophils, platelets), renal and liver function tests, blood chemistry, and C-reactive protein, were performed. Initial laboratory findings upon arrival are shown in Table 1.

**Table 1:** Summary of laboratory results at hospital admission in the patient with suspected acute generalized exanthematous pustulosis The results highlight leukocytosis with elevated neutrophils and eosinophils, as well as an increased C-reactive protein level.

Lab test	Result	Reference value
Hemoglobin	13.8 g/dL	13-18
Hematocrit	40.1%	12- 15
Leukocytes	18.4 x10⁹/L	4.60-10.20
Neutrophils	16.14 x10⁹/L	1.5-7
Lymphocytes	0.4 x10⁹/L	1-3.70
Monocytes	0.72 x10⁹/L	0-0.70
Eosinophils	0.75 x10⁹/L	0-0.40
Basophils	0.11 x10⁹/L	0-0.10
Platelets	165,000/mm³	150-450
Urea	19.7 mg/dL	16.6-48.5
Blood urea nitrogen	9.2 mg/dL	8.9-20-6
Creatinine	1.23 mg/dL	0.72-1.25
Total bilirubin	0.55 mg/dL	0.20-1.20
Alanine aminotransferase	42 U/L	0-55
Aspartate aminotransferase	26 U/L	5-34
Alkaline phosphatase	90 U/L	40-150
Lactate dehydrogenase	299 U/L	125-220
Gamma-glutamyl transferase	68 U/L	12-64
C-reactive protein	375 mg/dL	<1.0

Histopathological analysis of the skin biopsy (study number: 7570) reported epidermal spongiosis, subcorneal neutrophilic pustules, and marked dermal edema with scattered eosinophils, as well as a perivascular lymphoplasmacytic infiltrate, findings consistent with AGEP. Microscopic images are not available.

The patient received supportive dermatologic care, consisting of emollient and colloidal powder applications (10-15 minutes every 12 hours for seven days), petrolatum applied twice daily, and full-body oleo-calcareous liniment. Due to clinical stability, hospital discharge was decided. At follow-up one month after discharge, the dermatosis had resolved, leaving only post-inflammatory hyperpigmentation.

## Discussion

The incidence of AGEP is estimated at approximately one to five cases per million individuals annually. The condition is most frequently triggered by antibiotics, with β-lactams, β-lactamase inhibitors, cephalosporins, macrolides, fluoroquinolones, sulfonamides, and clindamycin being among the most commonly reported causative agents [6]. Following antibiotics, hydroxychloroquine has been identified as one of the most frequently associated medications with AGEP [7]. Several additional medications have been associated with AGEP (such as acetaminophen and nonsteroidal anti-inflammatory drugs (NSAIDs) like ibuprofen, piroxicam, and celecoxib). It is estimated that approximately 90% of AGEP cases are drug-induced, while the remaining instances are linked to viral or bacterial infections (*Chlamydia pneumoniae*, *Mycoplasma pneumoniae*, *Coccidioides* spp., SARS-CoV-2, cytomegalovirus (CMV), Epstein-Barr virus (EBV), and parvovirus B19). In children, viral infections are thought to represent a predominant cause of AGEP [3,6]. 

Following exposure to the causative agent, antigen-presenting cells activate drug-specific CD4 and CD8 T cells. These T cells migrate to the skin, where CD8 cells induce keratinocyte apoptosis via perforin/granzyme B and Fas ligand pathways, resulting in epidermal damage and vesicle formation. Early AGEP vesicles contain CD4 T cells and keratinocytes, which secrete high levels of CXCL8. This chemokine attracts neutrophils to the vesicles. The influx of neutrophils transforms the vesicles into sterile pustules [2]. 

Clinical presentation

Initial symptoms often include fever and malaise, typically accompanied by leukocytosis with marked neutrophilia. This systemic presentation is followed by the rapid onset of a pruritic pustular rash on a background of edematous erythema, predominantly affecting the trunk and flexural regions, while generally sparing the mucous membranes (mucosal involvement is seen in 20% to 25% of patients, although it is not a typical feature of AGEP and is usually confined to a single mucosal site). The pustules are numerous, sterile, and non-follicular in nature, and the eruption is commonly followed by superficial desquamation and collarettes of scales, finally resulting in hyperpigmentation [6,8,9].

In suspected or confirmed cases of AGEP, early laboratory evaluation is essential. Recommended tests include a complete blood count with differential (neutrophils, eosinophils, and platelets), renal and liver function tests, blood chemistry, and C-reactive protein. In patients with fever (≥38.3°C), blood cultures and pustule samples for bacterial and fungal analysis should also be obtained [1]. In a study of 58 AGEP patients, 17% showed systemic organ involvement, mainly affecting the liver, kidneys, and lungs. Hepatic involvement presented as elevated liver enzymes, sometimes with hepatomegaly or steatosis. Pulmonary issues included bilateral pleural effusions causing hypoxemia. High neutrophil counts and CRP were linked to systemic involvement [2,10].

The diagnosis of AGEP is based on a combination of clinical presentation and histological findings. In 2001, the European Study Group on Severe Cutaneous Adverse Reactions (EuroSCAR) introduced a scoring system with a maximum of 12 points that incorporates detailed aspects of morphology, histology, and the clinical progression. This tool helps categorize patients suspected of AGEP into definite, probable, possible, or unlikely cases (interpretation: 0 points indicate no AGEP; one to four points, possible; five to seven points, probable; and eight to 12 points, definite) [6, 11]. Based on the EuroSCAR scoring system, the patient in this case scored eight points, which classifies the diagnosis as definite AGEP.

A skin biopsy that includes a pustule is recommended in all AGEP cases to confirm the diagnosis and rule out other possible conditions [1]. Histological characteristics of AGEP include the presence of pustules located intra-corneally, subcorneally, and/or within the epidermis, accompanied by papillary dermal swelling and a perivascular and interstitial infiltrate composed of both neutrophils and eosinophils. The intraepidermal pustules are typically found in the upper layers of the epidermis and can be continuous with subcorneal pustules. Additionally, spongiosis is frequently observed, especially in the intracorneal and subcorneal pustules, and necrotic keratinocytes are commonly present as well [6]. 

Treatment

Hospital admission is advised, preferably in a dermatology or internal medicine unit, to ensure proper bed rest, supportive skin care, and close clinical and laboratory monitoring [1]. The key treatment for AGEP is immediate withdrawal of the causative drug. When triggered by infections or other causes, managing the underlying condition is essential. Systemic corticosteroids have been shown to shorten hospital stay in AGEP patients [6,12]. High-potency topical corticosteroids (such as clobetasol) are recommended at doses of 20-40 g per day. They should be applied once daily over the entire body, avoiding the face, which is typically unaffected in AGEP. A daily treatment for five to seven days is recommended, that is, until complete or almost complete clearing and/or post-inflammatory desquamation [1]. Usually, AGEP resolves within two weeks [8]. Given its benign, self-limiting nature, specific treatments, particularly systemic corticosteroids, are typically unnecessary [11].

## Conclusions

This case highlights the importance of considering AGEP in the differential diagnosis of acute febrile pustular eruptions, even without a history of drug allergies. The patient had previously taken oral ibuprofen without adverse effects, and although drug reactions are the most common cause of AGEP, no specific medication was identified as the trigger in this case. Based on the clinical presentation and epidemiology, an infectious etiology was suspected. Polymerase chain reaction (PCR) and cultures for infectious organisms were not performed; however, based on the clinical presentation, an upper respiratory tract infection was suspected. Diagnosis was confirmed through clinical, laboratory, and histopathological findings, enabling timely management. Literature supports that while drugs are the predominant cause, infections must also be considered. Although AGEP is usually self-limited, early identification and appropriate treatment of the cause are essential to prevent complications. Overall, this case underscores the value of an integrated diagnostic approach to optimize care in severe cutaneous adverse reactions.
